# Case Study—Spiking Neural Network Hardware System for Structural Health Monitoring

**DOI:** 10.3390/s20185126

**Published:** 2020-09-08

**Authors:** Lili Pang, Junxiu Liu, Jim Harkin, George Martin, Malachy McElholm, Aqib Javed, Liam McDaid

**Affiliations:** 1 Industrial Center/School of Innovation and Entrepreneurship, Nanjing Institute of Technology, Nanjing 211167, China; 2 School of Computing, Engineering and Intelligent Systems, Ulster University, Derry BT48 7JL, UK; jg.harkin@ulster.ac.uk (J.H.); gs.martin@ulster.ac.uk (G.M.); m.mcelholm@ulster.ac.uk (M.M.); javed-a@ulster.ac.uk (A.J.); lj.mcdaid@ulster.ac.uk (L.M.)

**Keywords:** structural health monitoring, damage state classification, spiking neural networks, feature extraction, artificial neural networks

## Abstract

This case study provides feasibility analysis of adapting Spiking Neural Networks (SNN) based Structural Health Monitoring (SHM) system to explore low-cost solution for inspection of structural health of damaged buildings which survived after natural disaster that is, earthquakes or similar activities. Various techniques are used to detect the structural health status of a building for performance benchmarking, including different feature extraction methods and classification techniques (e.g., SNN, K-means and artificial neural network etc.). The SNN is utilized to process the sensory data generated from full-scale seven-story reinforced concrete building to verify the classification performances. Results show that the proposed SNN hardware has high classification accuracy, reliability, longevity and low hardware area overhead.

## 1. Introduction

Earthquake is an oscillatory movement caused by the abrupt release of strain energy stored in the rocks within the crust of earth surface. Areas are always vulnerable to natural disasters, which can lead to extreme damages in nearby populations in terms of fatality, communication and infrastructure loss. Flood, earthquake, cyclones and so forth, are among the most common occurring natural disasters across the world. The impact of these disasters differs in different geological and geographic locations. These disasters come with no advance warning but an effective, well prepared and maintained infrastructure will decrease the potential impact of future disasters. The structural health of buildings and other infrastructure suffers degradation due to environmental catastrophes caused by ageing, hazards and natural disasters [1]. In any area, public infrastructures, like schools, hospitals, fire stations, administrative buildings, bridges and treatment plants, are more prone to being highly affected by these disasters. Therefore, regular structural health monitoring is required to ensure the heath and endurance of these mega structures. In the event of a disaster, it is particularly important (i) to detect and quantify the severity of damage caused by environmental disasters at an early stage; (ii) to assess the current structural health and reliability of buildings to ensure their safe use; and (iii) to estimate repair costs for damage to minimize economic losses [2]. Traditional monitoring methods rely on an inspection and assessment of the buildings and requires experienced inspectors. Many structures are not convenient for on-site monitoring due to the terrain obstacles, that is, the lack of access to such buildings, which sometimes make it too late due to the retrospective nature of inspections [3]. An automated process such as installation of a Structural Health Monitoring (SHM) system for vulnerable structures, for example, buildings, bridges and even special launch vehicles, could periodically detect and notify of structural damages [4]. An advance SHM system should include the current health profile of the structure, the functions of damage detection, structural life prediction and so forth [5]. The lifespan of a typical structure lasts for decades whereas sensory instruments and microprocessors used by SHM systems come with a limited lifespan, for example, in an ideal operating environment the three-axis accelerometer of IIS3DHHC from the STMicroelectronics has a ten-year production life which shrinks in harsh outdoor environments. Therefore, after installation and regular use for several years, SHM systems may fatigue and fail. Due to technical and economic difficulties for secondary deployment, the longevity and reliability of SHM systems are key challenges that must be considered.

Considering these issues, SHM Systems should offer three characteristics. Firstly, the system should be adaptive, robust and capable of learning quickly. Secondly, the data analysis of the SHM system should be fast, efficient and accurate. Finally, the longevity and reliability of the systems hardware should be enhanced as the SHM system may be deployed in harsh conditions. The SHM system must have protection capabilities to resist the hazardous effect of the external environment. Recent research suggests that we can build a human brain-like, fault-tolerant, energy-efficient system with learning capability to enhance the robustness, productivity and endurance of the electronic hardware systems [6,7]. Spiking neural networks (SNN) are referred to as the 3rd generation of artificial neural networks (ANN). Contrary to conventional ANNs, SNNs are more realistic mathematical representations of the human brain that mimic biological spike-based event-driven processes to communicate between neurons. SNNs are more computationally complex and powerful than conventional ANNs [8]. On an embedded processor, these digital systems’ spike-driven communication capability makes SNNs, that is, the astrocyte-neural network model, more energy-efficient and reliable than deep neural networks [9]. Therefore, this paper proposes an SHM system that is based on SNN hardware to address the challenges of longevity and reliability of the monitoring system. The acceleration data collected from a full-scale seven-story reinforced concrete building are analyzed and the severity of the damage to the building is subsequently classified. The proposed system can monitor and detect the structural health damage levels under different environmental conditions and provide a high detection accuracy and relatively low hardware overheads for implementation.

The following section (Section 2) explores related work and briefly reviews the current SHM solutions and methodologies used to assess the structural health of buildings and structures. Section 3 defines the proposed SHM system and discusses feature analysis and classification methods for the sensor data. Section 4 provides the experimental results to demonstrate the feasibility and accuracy of the proposed system through actual building sensor data. Finally, Section 5 concludes the paper and gives the directions for future work.

## 2. Related Works

SHM systems need to provide a framework for damage classification using a continuous record of structural health monitoring data. This classification framework requires the categorization of many datasets relating to different states of structural health [10]. Damage identification in SHM involves four main steps—signal acquisition, signal processing, feature extraction and classification. The acquired data are then analyzed by signal processing techniques to extract, identify and classify key features which are used for assessing the health condition of the structure. Feature extraction and classification techniques are very critical for the assessment of the structural health condition in an automated system. The feature extraction method focuses on extracting features which may indicate a damage state ‘hidden’ in the recorded sensor data, for example, the orthogonal decomposition method is used for feature extraction and analysis [11]. Feature extraction relies on empirical data. As the structure is affected by environmental conditions, sensor data include noises which affect damage level assessment [12]. Therefore, feature extraction is a foremost and critical step for the SHM system.

Another challenge of SHM systems is the damage classification method. Previous research proposed various damage classification methods for different structures. Conventional classification methods include clustering algorithms [13], that is, k-means (KM), which is widely used in SHM. However, KM is sensitive to the extracted data features and the initial choice of cluster centers [14] that may lead to erroneous classifications [13]. ANNs have been shown to be a promising technique for SHM classification [9]. They include a set of computational models inspired by the interconnected neurological structure of the human brain for learning and solving problems such as pattern recognitions. Taking into account the different classification rules of different structures and the use of different types of sensors [15] (e.g., sensors for measuring mechanical properties [16,17] and sensors for measuring environmental properties [18,19,20]), neural networks have the ability to extract features from the data automatically [21], which can meet the requirements of applications. However, existing systems are not suitable for detecting and analyzing the structural characteristics in real applications such as SHM, as the system cannot meet practical needs in terms of hardware cost and power consumption.

Unlike traditional ANN, Spiking Neural Networks (SNNs) have a smaller hardware overhead and are more reliable and power efficient. It has been reported that SNN hardware, such as neuromorphic systems, consume two orders of magnitude less energy than ANNs [22]. In brain-inspired intelligence research, SNNs demonstrate a low power consumption and high performance for the deployment of artificial intelligence technology. In addition, if considering the glial cell, such as an astrocyte, spiking neural astrocyte networks have shown a self-repairing capability by using a novel learning rule [23]. Therefore, this work proposed an SHM solution based on a SNN hardware system with self-repairing capability that will improve the electronic system reliability and life-span in harsh environments. To the best of the authors’ knowledge, conventional ANN and Probabilistic Neural Networks (PNN) are widely used for structural damage detection [24,25,26] but no structural health monitoring application of SNN has been reported in the literature. Therefore, by combining the energy-efficient SNN classification algorithm and the highly compact neural network hardware, the performance and lifetime of the SHM system can be improved. The results in Section 4 will demonstrate that the proposed work makes SHM a viable option with low energy consumption, anti-noise capability and an efficient data processing capability.

## 3. SHM System Based on SNN

This section explores the architectural components of the proposed SNN based SHM system including data acquisition (sensors) and decision-making mechanism (damage level classification). Furthermore, benchmarks of K-means and ANN algorithms are also briefly introduced in this section.

### 3.1. System Architecture

**Figure 1:**
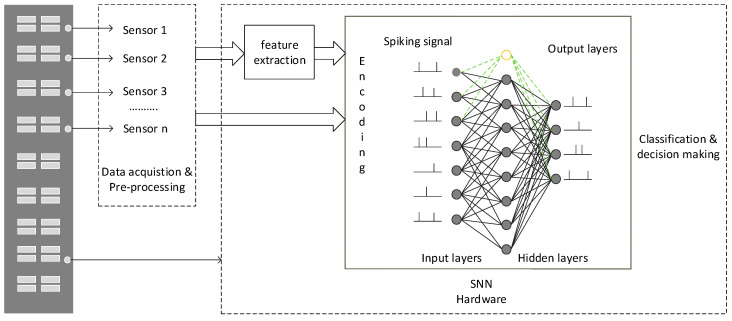
A Spiking Neural Network (SNN)-based Structural Health Monitoring (SHM) system.

SHM is a multi-layered hardware system that is comprised of multiple sensors for data acquisition, communication and processing architecture to assess the health of structural integrity. Figure 1 shows the structure of the proposed SHM system. The system is equipped with wired or wireless sensors, such as accelerometers, to collect the data from under observation structure. Through the analysis of the raw data, appropriate features can be selected and extracted from the time domain or frequency domain. After feature extraction, the data is fed into the SNN hardware system for the structure damage level assessment. The SNN encodes the pre-processed data into input spiking signals. This work proposed two SNN models to explore an efficient and cost-effective solution for the SHM system. A fully connected SNN network based on Leaky Integrate and Fire (LIF) neurons with SpikeProp as a learning algorithm for feature classification. The second model is based on the Neucube framework [27] using the Spike Timing Dependent Plasticity (STDP) rule for the unsupervised training and deSNN [28] algorithm for supervised learning. Both models can classify the level of structural damage to identify structural health status.

SNNs use time as an input dimension and record valuable information in a spatial domain. The information received by the spiking neuron is a pulsed time series, so the analogue sensory data needs to be encoded into the spatial dimension for input to the spiking neural network. The spiking neuron membrane changes upon arrival of input spike and each postsynaptic neuron fires an action potential or spike at the time when the membrane potential exceeds the firing threshold [29]. The event-driven neurons in an SNN are only active when they receive or emit spikes, which can contribute to energy efficiency over time.

Hardware systems that implement neuronal and synaptic computations through spike-driven communication may enable energy-efficient machine intelligence [30]. Compared with the traditional neuron model, the spiking neuron model has lower power consumption and is also suitable for parallel computing. Therefore, using a spiking neural hardware system can speed up the computation power.

### 3.2. Feature Extraction

Considering different sensors used in the structure, the selection of damage-sensitive features is generally based on multiple tests, so as to determine which features can indicate the health state of the structure accurately and are robust to the influence of the structural conditions and environments. These features can be extracted from the time domain (e.g., mean, variance, peak to peak amplitude, Zero crossing rate, energy, maximum amplitude, etc.) and frequency domain such as Fourier transform. Mean, variance and zero crossing rate are defined as:

(1) $$mean\left(a\right)=\frac{1}{N}\overset{N}{\underset{i=1}{\sum }}a_{i}$$

(2) $$variance\left(a\right)=\frac{1}{N}\overset{N}{\underset{i=1}{\sum }}{\left(a_{i}-mean\left(a\right)\right)}^{2}$$

(3) $$\;zcr\left(a\right)=\frac{1}{N-1}\sum_{i=1}^{N-1}\Pi \{a_{i}a_{i-1}<0\},\;\Pi \left\{A\right\}=\{\begin{array}{cc} 1 & A\;is\;true\\ 0 & A\;is\;faulse \end{array},$$

where *a* is the input sensor data, *N* is the number of the samples. After feature extraction, supervised or unsupervised learning methods can be used for data analysis and structure health status classification.

### 3.3. Structure Damage Classification

**Figure 2:**
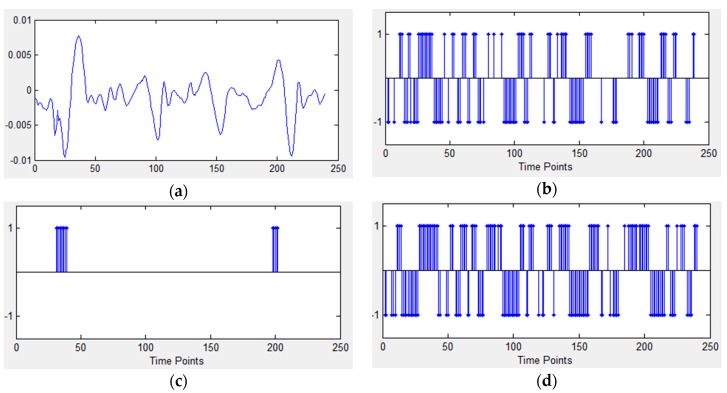
Spike trains generated by three different coding schemes. (**a**) Data stream of a channel; (**b**) Encoding with Address Event Representation (AER); (**c**) Encoding with Bens Spike Algorithm (BSA); (**d**) Encoding with Step Forward (SF). Note that spikes in (**b**), (**d**) are positive or negative but there are only positive spikes in (**c**).

Temporal coding schemes, such as Address Event Representation (AER), Bens Spike Algorithm (BSA) and Step Forward (SF), are used to represent information as an input to SNNs. Figure 2 shows different encoding results for the same temporal input data. The spike trains will carry the key information of the original signals. Different spike encoding algorithms have distinct characteristics when representing input data. BSA, shown in Figure 2c, is suitable for high frequency signals, so there are few spikes encoded from the low frequency signals, while AER and SF are better to represent the signal intensity.

Different spiking neuron models can be used to model spike generations at different description levels of biology [9], such as leaky integrate-and-fire (LIF), Izhikevich and Hodgkin–Huxley. The LIF neuron is one of the simplified models, which can be modelled as:

(4) $$\tau_{m}\frac{dV_{mem}}{dt}=-\left(V_{mem}-V_{eq}\right)+RI^{ext},$$

where $V_{mem}$ is the membrane potential of the neuron, $I^{ext}$ is the external driving current, $\tau_{m}$ is the membrane time constant, R is the input resistance and $V_{eq}$ is the equilibrium potential of the leakage conductance.

**Figure 3:**
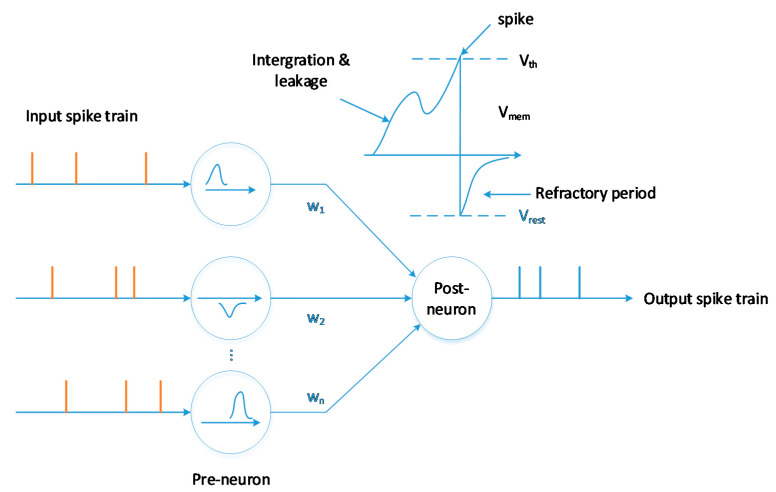
SNN neuron and computation model.

Figure 3 shows the state of the neuron updated by the membrane potential under the synaptic stimuli. When the membrane potential of the neuron crosses the threshold, the neuron then generates an output spike, which acts as an input stimulus for subsequent layer neurons.

SNN can be trained using unsupervised and supervised approaches. An unsupervised SNN, using the Spike Timing Dependent Plasticity (STDP) learning rule, was demonstrated with competitive accuracy [31]. The weight update in the STDP learning rule [32] can be described as:

(5) $$∆w=\{\begin{array}{c} \alpha_{+}e^{-∆t/\tau_{+}}\;\;\;\;\;∆t\geq 0\\ \alpha_{-}e^{∆t/\tau_{-}}\;\;\;\;\;∆t<0 \end{array},$$

where ∆w is the weight change rate, $\tau_{+}$ and $\tau_{-}$ are STDP time constants, $\alpha_{+}(>0)$ and $\alpha_{-}(<0)$ are constant coefficients and ∆t is the time difference between a post-neuron and a pre-neuron spike. When ∆t≥0, the synaptic plasticity is a long-term potentiation (LTP) process; otherwise it is a long-term depression process. Two different SNN structures are adopted in this study, where one is a fully connected SNN and the other one is a model based on NeuCube [27].

For performance comparisons, the commonly used classification algorithms of K-means and ANNs are also used in this work for benchmarking. A supervised learning algorithm of ANN is used in this work, where the network weights are adjusted in every iteration by comparing the difference between actual output and the targeted output. A multi-layer feedforward architecture with input layer for sensory input, hidden layer for learning and an output layer to generate spiking output. The number of input neurons equals the number of sensors, whereas the output layer neurons represent the number of structure level classifiers. For K-means, the unsupervised K-means algorithm for SHM can be described with the following steps, where k is the number of desired clusters—(a) Given the features’ matrix as an input, find the k centroids (random or select); (b) Calculate the distances between features’ vectors and centroids; (c) Group the features’ vectors based on their intra-cluster distance; and (d) Iterate the algorithm and update the centroids for a better clustering result.

## 4. Experiments

This section explains experimental setup to generate damage level report for SHM system. Furthermore, this case study analyses and compares the results of three classification methods, K-means, ANN and SNN to identify the best performing SHM system.

### 4.1. Dataset

This case study used a full-scale seven-story reinforced concrete building dataset for experimentation [1]. The building is installed with 45 accelerometers operating at a sampling rate of 240 Hz. A sequence of dynamic tests was applied to the building over several months, including ambient vibration tests, free vibration tests and forced vibration tests using the shake table of Network for Earthquake Engineering Simulation at University of California, San Diego (UCSD-NEES). A 0.03 g root-mean-square (RMS) acceleration white noise base excitation and ambient vibration tests were performed on the structure before and between earthquake shake-table tests. For 45 channels, the signal to noise ratios (SNR) are −36.97 db~22.81 db. The building was damaged progressively through several historical earthquake ground motions and damage states of the building can be described as shown in Table 1. In 1st to 3rd earthquakes, the roof drift ratio, defined as the ratio between the maximum lateral displacement at the roof level of the building and the height of the roof relative to the base of the building, was measured as 0.28%, 0.75% and 0.83%, respectively. The maximum tensile strain in the longitudinal reinforcing steel was measured close to the base of the wall as 0.61%, 1.73% and 1.78%, respectively [1].

### 4.2. Feature Extraction

**Figure 4:**
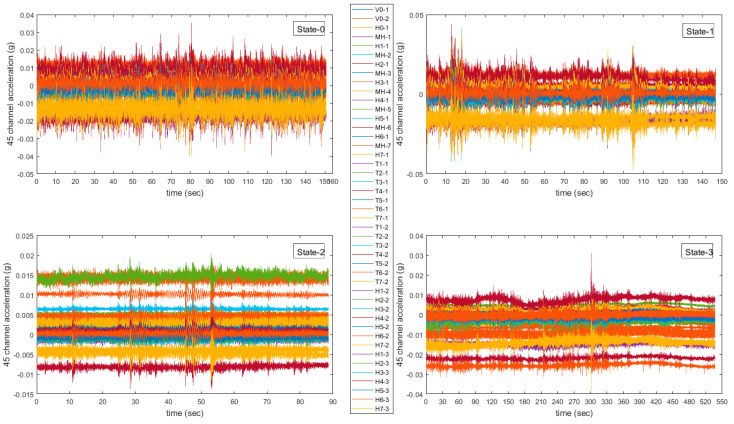
Row data from 45-channel accelerometers.

The raw data collected from 45 channels in the building at different health states are shown in Figure 4. Raw accelerometer data of different structure states show different features, such as maximum amplitude and mean value and so forth. By considering the building’s physical movements in different states [33], the deformation degree of buildings can result in large differences in the mean and fluctuation range of the accelerometer data. Based on this analysis, zero-crossing rate, mean and variance are used for feature extractions.

**Figure 5:**
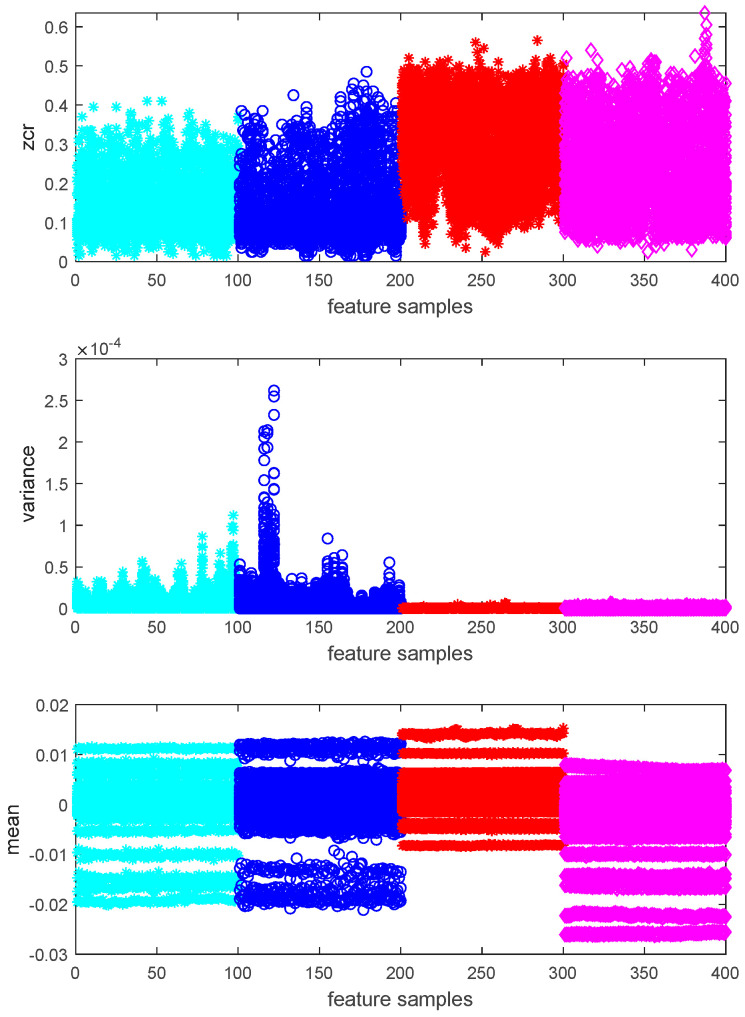
Results of the features extracted from raw data.

After the data have been pre-processed, three methods (including zero-crossing rate, variance and mean value) are used to extract data in order to select the damage-sensitive features. The features are presented in Figure 5. The zero-crossing rate, which is the rate of sign-changes along a signal, is too weak to separate the different damage states (indicated by colors). Among them, calculating the mean value of the sensor data has the potential to differentiate the four damage states.

### 4.3. SHM Classification Results

For different classification methods, 70~80% samples (including mean samples and raw data) are used for training and the rest for validation and testing.

#### 4.3.1. K-Means

A 50 step-length sliding window with 100 sample points is used to get more mean samples, which are used as an input for the k-means algorithm. K-means parameters are shown in Table 2.

**Figure 6:**
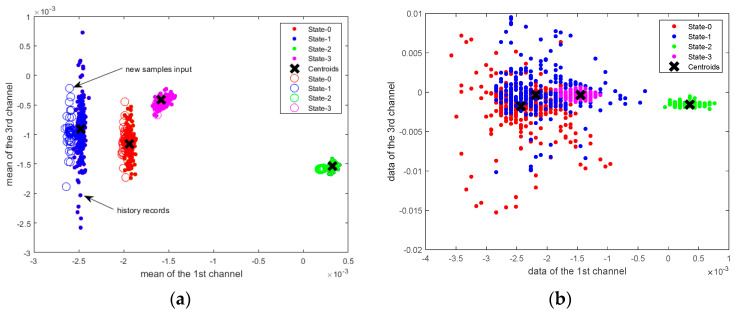
SHM classification using K-means. (**a**) Clustering of mean samples; (**b**) Clustering of raw data.

It can be seen from Figure 6a that using the mean value of the data as an input of the k-means algorithm can classify the health status of the building. The dots represent historical records and the circles represent new data inputs. The classification accuracy of the structural health status is 100%. In Figure 6b, the raw data are used directly as the input of the k-means algorithm. In the case of overlapped data, including State-0, State-1 and State-3, the k-means algorithm cannot separate these data. There are 45 channels in total and only two of them are used for the demonstration in Figure 6.

By incorporating the hardware design process [34] to implement K-means, the input data dimension area will be about 3.46 mm^2^ and 1.23 mm^2^ for the parallel mode and multiplexed architecture, respectively.

#### 4.3.2. ANN

The ANN with 45 input neurons, 20 hidden neurons and 4 output neurons can get similar accuracy with different input samples (mean samples and raw data). Table 3 shows that ANN slightly confuse between State-0 and State-1 when trained on raw data samples. The hardware area of the neuron is estimated as about 1.347 mm^2^ based on a 45 nm CMOS technology [35]. It can also be calculated from Reference [36] that the total hardware area of the ANN is >0.798 mm^2^.

#### 4.3.3. NeuCube

**Figure 7:**
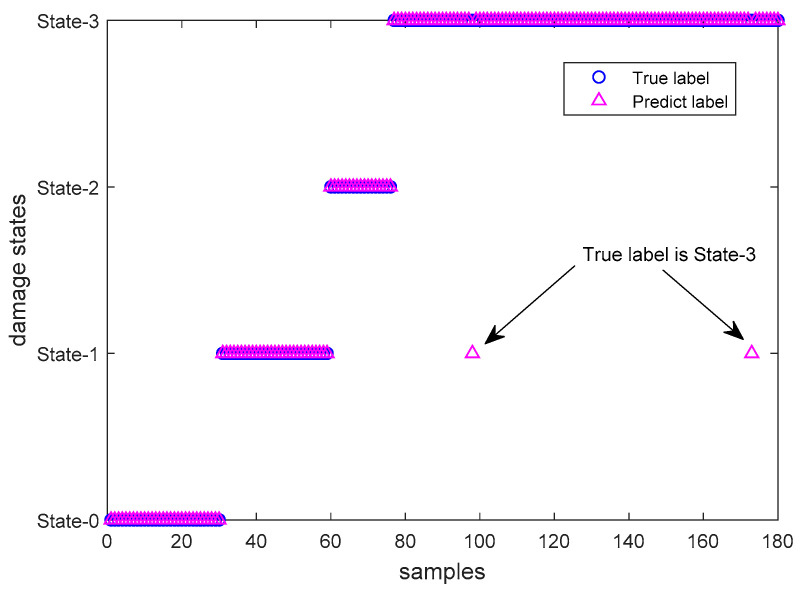
Classification result by using NeuCube (raw data).

In NeuCube, raw data samples are fed into a dynamic SNN. One channel of an input sample was shown in Figure 2a. Table 4 shows network parameters used by NeuCube. The model is established with 45 input neurons, 50 hidden neurons and output neurons (the number of samples). Due to the dynamic structure, the overall area overhead of NeuCube SNN is about 4.655 × 10^−3^ mm^2^ is calculated according to the neuronal and synaptic hardware area estimation proposed in References [37,38]. Results shows that overall classification accuracy of NeuCube SNN is 98.9% (as shown in Figure 7).

Table 5 shows the breakdown of performance accuracy for the classification of damage states observed by NeuCube. Enough samples will contribute to a higher probability of making the correct decision about the damage states. As a comparison, mean samples are input into NeuCube with the same parameter settings above. The accuracy is not as stable as raw data input, as NeuCube is more sensitive to temporal raw data [39].

#### 4.3.4. Customized SNN

A customized fully connected SNN with LIF neurons and SpikeProp as a learning algorithm is developed for the SHM classification based on previous work [40]. The three-layered fully connected SNN is designed and modelled in MATLAB. Table 6 shows network topology, size and hardware area of the LIF based SNN model. Mean sensory samples are fed through 45 spiking input neurons to propagate spike towards 10 hidden neurons in order to generate 4 state output at 1 output neuron. The estimated hardware area of the SNN chip shown in Table 6 is calculated using References [37,38].

Damage states are encoded with the time of spike of output neuron (SNN output). The experimentation results show the classification accuracy using the mean samples input. The results show in Table 7 that the proposed customized SNN classifies the structural damage with 99.18% accuracy for the mean dataset. Moreover, the overall accuracy can be higher, up to 99.46%, by increasing the number of iterations, as compared to the 98.9% NeuCube average accuracy for raw sensory input.

#### 4.3.5. Discussions

A summary of results using K-means, ANN and SNN in SHM applications, is shown in Table 8. ANN used raw data and feature samples as an input and there is little difference in classification accuracy. The final decision making can be the same within a certain confidence interval. Thus, if ANN combines the feature extraction into the learning process, it improves the computing speed and also reduces the hardware consumption. The structural damage occurrence detection can be assessed as health (State-0) and damage (State-1, State-2 & State-3), then the sensitivity (true positive rate) and specificity (true negative rate) of three typical methods can be obtained with the input of raw data samples, as shown in Table 9. Compared with the other two algorithms, SNN can accurately determine whether the structure is healthy. Meanwhile, the hardware area consumption of SNN is much less than ANN, the classification accuracy has a little difference of 0.9% and the sensitivity and specificity are higher. In summary, the proposed method based on SNNs apparently achieves a good trade-off between classification, reliability and hardware resource consumption.

## 5. Conclusions

The structural health state detection in this study involves the feature extraction from periodic observation measurements of a structure, where these features are analyzed to determine the current health state of the structure. Based on the detected states, the appropriate repair and strengthening of structures can keep the structure operational and longeval. Through the analysis of ZCR, Mean and Variance of the raw sensor data, it is found by experiments that the mean value is more sensitive to the structure state. Therefore, mean values and raw data were used as inputs and several classification methods, including K-means, conventional ANN and SNN, were used to detect the health state of the structure. Analysis and comparison results show that the SNN algorithm proposed in this study has advantages including (a) High classification accuracy can be obtained by directly using the raw data as input without manual feature extraction; (b) The small part of misclassification (1.92%) only exists in State-3, where the output health states can be clearly distinguished; (c) The hardware area of SNN is lower compared to ANN or K-means. In summary, the proposed SNN hardware solution for SHM has a stronger survivability and reliability than conventional approaches. Further work will further optimize the SNN for SHM systems in two respects including (a) to develop multi-layer (deep) SNNs to improve the accuracy and (b) to further analyze the sensor data to enhance the system functionalities, such as reporting the location of damage or life forecast of the structure.

## Figures and Tables

**Table 1:** Dynamic tests used in this study.

Damage State	Description
State-0	8 min white noise base excitation process & 3 min ambient vibration
State-1	After the 1st earthquake excitation, with 8 min white noise base excitation process & 3 min ambient vibration
State-2	After the 2nd earthquake excitation, with 8 min white noise base excitation process & 3 min ambient vibration
State-3	After the 3rd earthquake excitation, with 8 min white noise base excitation process & 3 min ambient vibration

**Table 2:** Parameters in k-means.

**Parameters Setting**	**Cluster Number**	**Distance**	**Initial Centroid Positions**	**Replicates**
	4	L1 distance	Random	8

**Table 3:** Classification matching matrix with different input samples.

	Predict Label	State-0	State-1	State-2	State-3
True Label					
(**a**) Mean samples					
State-0		100%	0.0%	0.0%	0.0%
State-1		0.0%	100%	0.0%	0.0%
State-2		0.0%	0.0%	100%	0.0%
State-3		0.0%	0.0%	0.0%	100%
(**b**) Raw data					
State-0		99.7%	0.3%	0.0%	0.0%
State-1		0.9%	99.1%	0.0%	0.0%
State-2		0.0%	0.0%	100%	0.0%
State-3		0.0%	0.0%	0.0%	100%

**Table 4:** NeuCube Model Parameter Setting.

Parameter		Description	Value
STDP Rate		Defines the learning rate of the STDP learning	0.01
Firing threshold		Defines the threshold membrane potential beyond which the neuron fires a spike.	0.5
deSNN Classifier Parameters	Mod	The weight is calculated as a modulation factor (the variable mod) to the power of the order of the incoming spikes.	0.55–0.6
	Drift	Initial connection weights are further modified to reflect the following spikes, using a drift parameter.	0.015

**Table 5:** Accuracy of each class.

Damage State	Accuracy
State-0	100%
State-1	100%
State-2	100%
State-3	98.08%

**Table 6:** SNN setting and result (mean samples).

Network.	Topology	Multiplier of Synapses	Total Neurons	Total Synapses
SNN	[45:10:1]	10	56	460
**Area of Neurons**	**Area of Synapses**	**Area Overhead**	**Overall Accuracy**	**Number of Iterations**
5.04 × 10^−4^ mm^2^	1.10 × 10^−3^ mm^2^	1.61 × 10^−3^ mm^2^	99.18%	2500
			99.46%	3000

**Table 7:** Accuracy of each class.

Damage State	SNN Output	Accuracy	
State-0	16	100%	100%
State-1	18	95.67%	97%
State-2	20	100%	100%
State-3	22	99.8%	99.9%
**Overall accuracy**		99.18%	99.46%

**Table 8:** Performance comparison of three methods in SHM application.

Method	Classification Accuracy		Technology	Hardware Area
	Raw Data	Feature		
K-means	80%	100%	TSMC 90 nm	1.23 mm^2^~3.46 mm^2^
ANN	99.8%	100%	CMOS 45 nm	1.347 mm^2^ (neurons only)
SNN	98.9%	99.46%	CMOS 90 nm	4.655 × 10^−3^ mm^2^ (NeuCube)
				1.61 × 10^−3^ mm^2^ (Customized SNN)

**Table 9:** Sensitivity and specificity comparison of three methods.

Method	Sensitivity	Specificity
K-means	92.97%	73.87%
ANN	99.94%	99.15%
SNN	100%	100%
